# Supplementary material for: The genetic basis of a social polymorphism in halictid bees
Source: Nat Commun. 2018 Oct 18;9:4338. doi: 10.1038/s41467-018-06824-8 (PMC6194137; doi:10.1038/s41467-018-06824-8)
Supplement: Supplementary file 1 — Supplementary Information [file 41467_2018_6824_MOESM1_ESM.pdf]

Supplementary Information

The genetic basis of a social polymorphism in halictid bees

Kocher *et al.*

Supplementary Tables 1-5
Supplementary Figures 1-6

Supplementary Table 1. Nucleotide diversity within each *L. albipes* population sampled. N=the number of individuals from each population; and pi is the estimated per-site nucleotide diversity for each population.

Population	Behavior	N	pi
All	Polymorphic	143	0.0020
AUD	Social	26	0.0010
DOR	Social	22	0.0016
RIM	Social	23	0.0013
BRS	Solitary	25	0.0011
VEN	Solitary	24	0.0009
VOS	Solitary	23	0.0013

Supplementary Table 2. Missense SNPs associated with the social polymorphism. Columns are as follows: SNP name, number of ungenotyped individual, the alternate allele, reference allele, allele frequency, p-value, FDR-corrected p-value, log(10) of the FDR corrected p-value, beta (as estimated by GEMMA), SE (as estimated by GEMMA), FST, frequency of the alternate allele in social populations, frequency of alternate allele in solitary populations, the gene ID in the current genome assembly, and the SwissProt best match.

SNP	N_missing	Alt	Ref	Allele freq	p_wald	fdr	log10(fdr)	beta	se	FST	Social a1 freq	Solitary a1 freq	Lalb_v3 OGS	SwissProt
LALB_1:12843152	1	G	C	0.232	1.41E-14	9.40E-10	9.03	-0.3499894	0.04070896	0.335112	0.428571	0.042254	LALB_03181	SYNE1_HUMAN
LALB_1:12843643	3	T	C	0.336	6.82E-11	1.48E-06	5.83	-0.2897452	0.04101899	0.322298	0.550725	0.128571	LALB_03181	SYNE1_HUMAN
LALB_1:12844541	2	A	C	0.206	2.09E-15	1.70E-10	9.77	-0.3523219	0.03945583	0.36016	0.400000	0.014286	LALB_03181	SYNE1_HUMAN
LALB_1:12844789	0	A	G	0.231	4.48E-25	1.76E-19	18.75	-0.4490689	0.03540324	0.413414	0.450704	0.014085	LALB_03181	SYNE1_HUMAN
LALB_1:12845893	0	C	G	0.154	4.72E-20	1.02E-14	13.99	0.4092384	0.03807738	0.207461	0.028169	0.281690	LALB_03181	SYNE1_HUMAN
LALB_1:12847652	1	A	G	0.12	2.65E-13	1.30E-08	7.88	0.3482799	0.0431103	0.184137	0.014085	0.228571	LALB_03181	SYNE1_HUMAN
LALB_1:12847976	1	A	C	0.12	7.13E-13	3.18E-08	7.50	0.3395468	0.0429741	0.178453	0.014286	0.225352	LALB_03181	SYNE1_HUMAN
LALB_8:10925589	1	G	T	0.225	2.71E-09	3.43E-05	4.47	-0.2576752	0.04055162	0.198615	0.371429	0.084507	LALB_06150	TRAP1_MOUSE
LALB_28:254477	0	C	T	0.14	2.89E-27	1.71E-21	20.77	0.4774595	0.03528001	0.271429	0.000000	0.281690	LALB_09027	CSK2A_SPOFR
LALB_30:229396	1	T	C	0.099	1.43E-11	4.18E-07	6.38	0.3421807	0.04652628	0.184499	0.000000	0.197183	LALB_08459	VAS1_BOVIN
LALB_30:229441	3	G	A	0.236	1.08E-25	5.12E-20	19.29	-0.4549382	0.03520425	0.417511	0.457143	0.014493	LALB_08459	VAS1_BOVIN
LALB_146:24619	0	A	G	0.091	9.11E-11	1.91E-06	5.72	0.3287797	0.04690836	0.171429	0.000000	0.183099	LALB_12465	MOS1T_DROMA
LALB_199:95760	1	C	G	0.134	2.78E-17	4.97E-12	11.30	0.368693	0.0381251	0.255664	0.000000	0.267606	LALB_12953	.
LALB_199:95811	1	A	G	0.218	4.61E-12	1.56E-07	6.81	-0.3096425	0.04094239	0.304561	0.400000	0.042254	LALB_12953	.
LALB_211:27749	3	C	G	0.157	8.61E-11	1.83E-06	5.74	0.2841674	0.04048122	0.120153	0.058824	0.253521	LALB_12690	BICD_DROME
LALB_211:28126	0	C	G	0.161	1.47E-10	2.76E-06	5.56	0.2799717	0.04046683	0.103687	0.070423	0.253521	LALB_12690	BICD_DROME
LALB_845:4616	4	T	C	0.223	4.17E-11	9.66E-07	6.01	-0.2935571	0.04102273	0.307055	0.405797	0.043478	LALB_14827	.

Supplementary Table 3. *L. albipes* candidate genes and the human orthologs associated with autism-spectrum disorder.

Lalb_v3	Human_gene_symbol	Swiss-Prot
LALB_02231	ASS1	ASSY_XENTR Argininosuccinate synthase
LALB_03181	SYNE1	SYNE1_HUMAN Nesprin-1
LALB_07179	SDK1	SDK1_MOUSE Protein sidekick-1
LALB_09030	STX1A	STX1A_DROME Syntaxin-1A
LALB_12523	DGKZ	DGK2_DROME Eye-specific diacylglycerol kinase
LALB_13718	CNKSR2	CNKR3_RAT Connector enhancer of kinase suppressor of ras 3

Supplementary Table 4. Significant SNPs associated with the *syntaxin 1a* locus. Columns as in Supplementary Table 2. Location denotes whether the variant was upstream or intronic in *syx1a.*

SNP	N_missing	Alt	Ref	Allele freq	Beta	SE	p_wald	fdr	-log10FDR	FST	Location
**LALB_28:262159***	**1**	**T**	**C**	**0.218**	**-0.354**	**0.037**	**3.22E-17**	**5.08E-12**	**11.29**	**0.382**	upstream
LALB_28:263596	0	T	C	0.14	0.477	0.035	2.89E-27	1.71E-21	20.77	0.271	upstream
LALB_28:265348	0	C	T	0.175	0.337	0.036	2.06E-16	2.56E-11	10.59	0.209	intron
LALB_28:265384	0	C	G	0.14	0.477	0.035	2.89E-27	1.71E-21	20.77	0.271	intron
**LALB_28:266299****	**0**	**T**	**C**	**0.21**	**-0.339**	**0.038**	**1.18E-15**	**1.00E-10**	**10.00**	**0.369**	intron
LALB_28:267669	2	A	C	0.121	0.347	0.043	1.87E-13	9.60E-09	8.02	0.188	intron
LALB_28:268471	6	A	G	0.109	0.313	0.045	1.60E-10	2.96E-06	5.53	0.163	intron

*SNP1; **SNP2

Supplementary Table 5. Primer sets used in this study.

Target	Product size (bp)	Forward	Reverse
COI	899	CAACATTTATTTTGATTTTTTGG	TCCAATGCACTAATCTGCCATATT
Syx1a SNP1: LALB_28:262159	866	TTTGGGCCTGTGTGTTTGTA	GCTACCAGAGGACGACGAAG
Syx1a SNP2: LALB_28:266299	790	TTGTTATGATTCCCCGTGGT	CTGCCGGTACTCTCGTTCTC
Syx1a qRT-PCR primer	111	GGATAGATTAGCAGCCCTCGT	TCCGCGAAAAACTCTGTCAT
Rps18 qRT-PCR primer	126	CGATTAAAGGTGTCGGTCGT	GGTTGCCCATAATGGTGAC
GAPDH qRT-PCR primer	184	CAGGTTGTTGCCATTAACGA	CTTTTCCCCAAGGAATGGAT

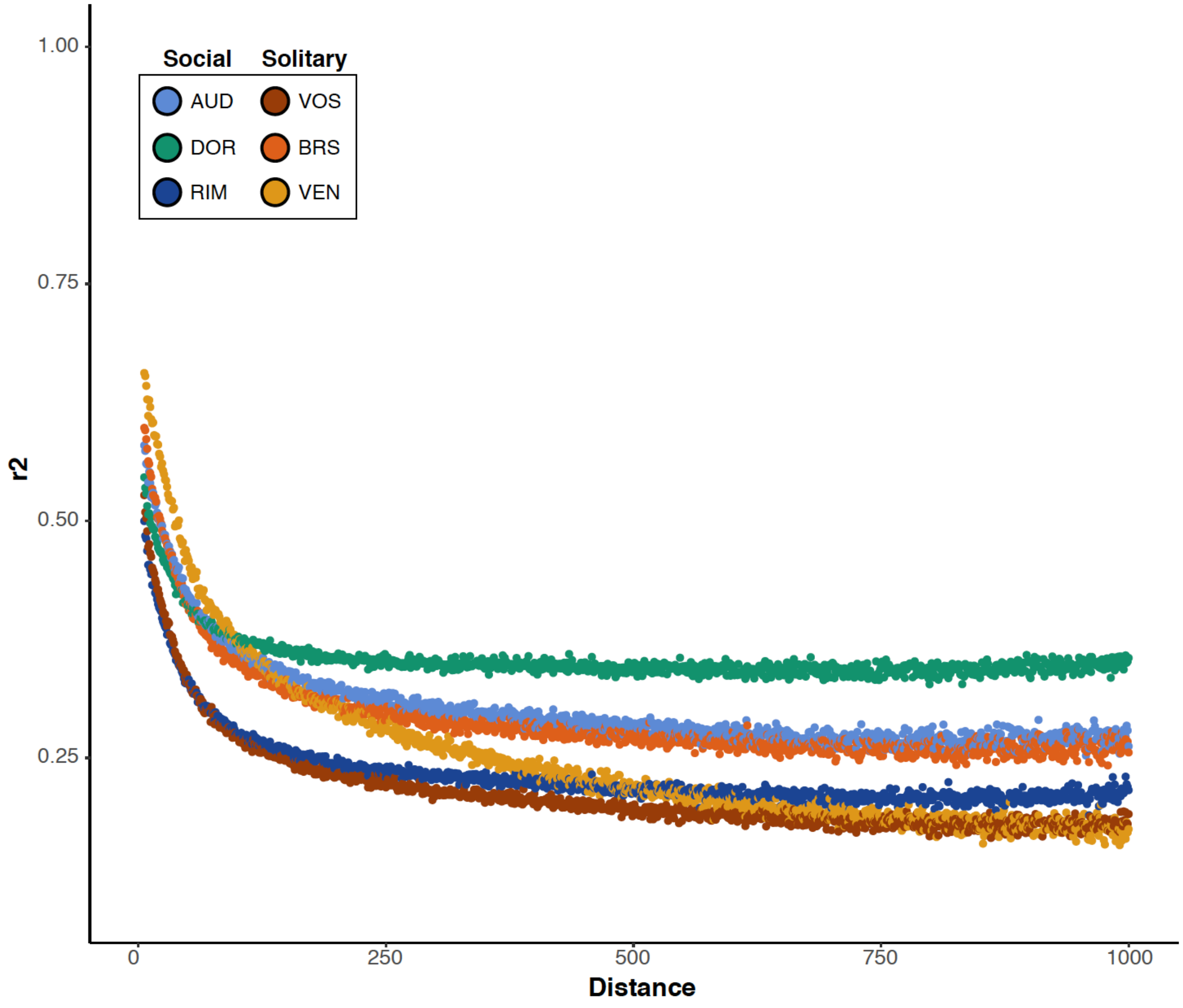

Supplementary Figure 1. Linkage disequilibrium decay plots for each of six *L. albipes* population sampled.

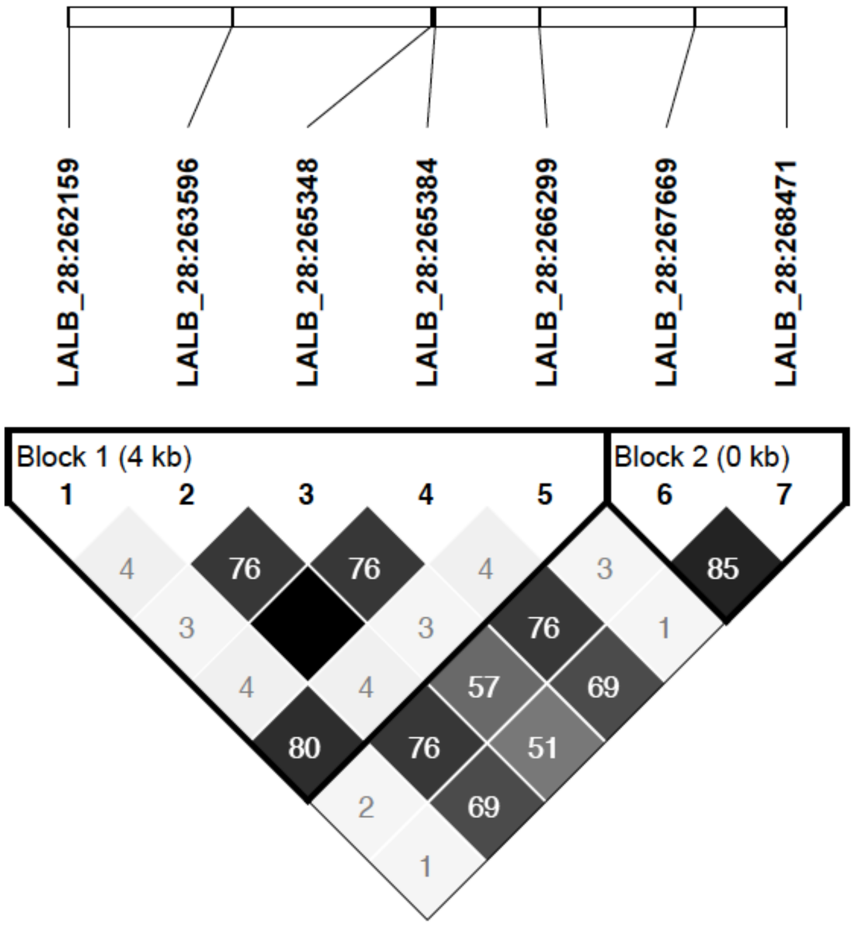

Supplementary Figure 2. Linkage disequilibrium plot for the 7 SNPs associated with *syx1a*. Values in each box are r^2 measures for each SNP pair.

>LALB_28:261159-265725

GTCAATTTGGCGTACACCTTTCACCGTGTACTTAGTAGTTAAGTATTTTCATTTTCGTCCGTGATAAATCGGAGGAATCGAGGA
GAAGAAAGCATGTGAGACGCTTAGCCTCCCAACGGAACTAAACGGAAAAAACAAACGGTTTTGTATAGAAAAGAAAAACACTG
TTTATATAATTTAAGTCGAACAAATCATACATTCACCTTCGCTTATAAAAATAGTAAACAGTCCTTATTATCGTGGACAGGCGCA
ACTGACTACAAACCGTGCTTCATCTCAATCGTAACATTATTTCAAAATACAACAGTATGTCTGGGAGTCGCAGATATTTCGCAG
AGACGATTGTTGTTGCACCGTTCGTTTGGCGAGTAATAATGTACATATTGATGCCCTCTTTCCTTTTTCCAATTCATTCGTCATC
GCGTAACCGAGGTGTAAAGTAACATCGAAGAGTATCGATTGATAAGGATTCGACTATTTTAAGAACTTTTGTTCCGGAGTGATG
TGTATTCAATTAAAAACTTTTGGGCCTGTGTGTTTGTATACGTTTCGATCTCCGTGTGAGTGTTTATGTGTGTATAAGTAAACAG
TGACTTCTAAAAATCAGCGTTTTTAGTTGTATCGATAGATTCTTTCTTGCAGAATTAATCCTTTACATTCTACATTCGTGATTTAC
GAAACAGCACGTTCATTCATTCGAAGATTCGACAGTTTTCTTTTTTTATTCATACTTTTTACTGATTTTAAGTAAGCTGCGACAGT
ATCGACAATATAAATGTATTTACTACCACGGTATATTTGAAATACGGTAGGCGATCTTGTTCACCAAACAAACAATTTCTTCGGT
TACGTTAAATCCATCGTGTCAGACAACATACGTTTATTCACGCATACACATTTACAATACGTAAGGGGGGCGTAAAGAATGTAG
AACAAGCACAACGTCGATCTCAGAACGACTTTACCTTCTCAACATGTAAAATTTTGAAAATTGTTACA[C/T]ATGGATTTTTAGCC
CTACATGGGTTCGTACCATATTTCGATATACGGCTTTACGTTATGCTTGGGTCATTCGGTACTGGTATAAAATTAGAGGTCTCG
CGTGCCACGTTCCCGCACACAGTGCGTTCGGAAATGCGGCGACGTTCAATTCCTGTATCCCTTCTTCAAGATAAAGCTCGCGT
GAAGTTTGAAATCACAGAAACATTTTTATCATTGTTAACGTATGGAACAAAATTTGACGCGAATTTTAATTCCTTCAAAGAAAGAA
TCTTCGACCTATAGGAGAGGCATGAGAACGCAACAGTCGAGGTACGCTCGTGTACGGTATGCCTCTGTTTGGATCGTGTGCA
CGATGTAACAAACTAAAGGTCTTCGTCGTCCTCTGGTAGCGCGGTTTCCTGTGCTTCCTTGAGGTCCTTTTCTATCTGTTGTTG
CCATTGTGGGTCCATGGTCACCTCGGGTGGCAAGAGAGCAGGCATGGCGACGAATTCCAAGTTTGGGTCGCCGATCAGCTTG
CGCGCCAACCACAAGAAAGGTTTCTCAAAGTTGTAATTGCTTTTCGCGCTGATATCATAGTACTAAAAACATGAAAAACATTGC
GATTAGTAAATTCGACGTATGCACAATTGCAAAATGAGTTGCACGGATTCGTCAGATTCTCCTAATTACCTGTAAATTCTTCTTC
CTGTGGAATATGATGCTCTTCGCTTTGACTTTTCTGTCCTTGATGTCGACTTTGTTGCCGCAGAGTACTATAGGTATGTTCTCG
CATACTCTTACCAAGTCTCTGTGCCAGTTCGGCACGTTCTTATATGTAACTCTTGATGTAACGTCGAACATGATAACAGCGCAT
TGTCCCTGAATGTAGTAACCGTCCCTAAGACCGCCGAATTTCTCTTGGCCAGCTGTATCCCAAACGTTGAACCTGATTGGCCC
TCGGTTCGTATGGAATATGAGAGGATGTACTTCGACTCCTAAGGTGGCGACGTATTTCTTCTCGAACTCGCCGGTTAAATGTC
GCTTGACAAACGTTGTTTTTCCAGTACCACCGTCCCCGACCAGGACACACTTGAAAGTCGGAATAGTTGCGTTCTGTGTCATTT
CGAGTGCCATGTTTGCTGAAGCGACGGATCTGAAAAATTAACGACGACCATGTGAATGTGAACAGGAAGGCCGCTGCTCGCG
GCAAAATTCACGCTCCGGGGATACCTCGTGAACAGAAACACTTTGCAACGGTTACAACGCGGTTTAGATCTCGCTAGATCACG
ATTACCGAGCGATTCCGCTATCGCGCGCGCGACAACCGAATCTTTCCTGCTTGTTAATTTTGGAAATGACTGGAAAGTACTAGT
TCTTGTTGGACGTATATCACGCGTCGGAAACGTGATCGGCCGCTATTCGTCGAACAACGGGGCCCGAAGAAACTGAAACGCG
TC[T/C]CGATTTACTTAAGATGTTTTGTCTATCGGATTCTCGATCGAATATACTTCGGCGAGCAGTACAAAAAGCCGGTTCGGCG
ATATTGCGTGACTATACCTGACGAGAAAAAATTCACCCACCACGTTCTCGGAGAATGGCGCCTCCCGTCGAAACACAGAGAAC
CACTTTTACGTTATACTCTATCGAAATACTATTATCGCTGGCGTTGCGGGAACGATGAACGGTTTAACAATCGAATGATTTGTCT
TTAAGCTCTGACGAGATTTTCAAAAATTGAACGGAGCGAGTACGAAATTTCCCGGCAAGTGACGTGTACGTGAAAATGACCGA
TAAAACTTACCCTGCGACACAGTTGTTATGGCTCACAATGGCGTTCCCTTGACTCGACGAGTAGGGGTCCCCTTTTTCGTCATT
CGTCGAGCACAACCAGGCTCGAGTTAAAGCGAACGTCTGTCCAATCAGTTCAGAAAAAATTCAATTTGAATCGTTTTCTCATTC
GATTGTTAAAATAATCCGTGGATCGTTGTGTTTTTTTGAAAATTCTAACAGGAATACGGCCGAACGTAGCGGCATCTATTTTATG
CGCGCCATATCGAACCGACGCCGATGAATTTTCGCTTTCAACGAAAGAACAGCGTCCTAACGGTAACTGCGCTCCTCCCTTCC
AATCAGAGAGCTTTCAGCCGATTCAGAAAGATGCCTCTCTTCAGTTGCGATAGTCAATGGTGTACCGCATACGATGCAATTCCA
ATGATCGCGTACTCGAACATGCTCACAATAAAGATTACAGAAGCGCCGAATTATTTAGCAACAGTGAGTCACGAGGTACGTTG
TAAATTGACATTCAACTTGATTAAGTGTGATTAAGTGTTACTAATCTAGAGCCGCATTATCAGTATCATGTCAAAACGCGTACAG
GTTTTATTCAAATTGAAAACCGTTACCATAAACATCAGTTTCATTCAAAGTTTTTGATTCAAAGCTTCAGATCTGCATTGGTTCAT
TATACTCAGAAATATTCTTTTCCTTAATACTACTGCTCCTTAAAAATTGCAGTATAACAGAAAGTTACAATAACTAGATTTATAGA
ACTCTCGTTAGATAGTTGCAATTAAATTGTGTAAGTTTAAAAGTCAAAAAGTGTGTAAGTTTTGACGGTCATTTGAAAGCAACAG
TTAGTCATACGAAATTACAAGATGGCGCCCTCCCGCGAAGAAACCGTAAAACTCCTCGAAACCAAATCCTCGGTAGCAGTTTC
AGGGTTTCGCCGTAGATGGCGACAAACGTGGTTTTTTACCGCATGGATCGAGTGCAGAGAAATTAAATTTTTAGGAGAACTGG
TGGATTGAACAATTGTTAACGAACGATAACTGACCAGCGAATACAATTTAGTATTGTTGTAGAAATACCGAGAGGTAGCATGCA
CGATTTCCTTTACATCCTCGTTTATAAAGCACCATCGAGTACCGTAACATACGAGAGAGGTGTTTTCTCATTATTTCTGCATTTC
TTGAACGTGTAGCGGCGGCAAATATGAAATTTTTTATCCTACATACTTATACGACTTATATCGTTCGGTTAGTATCTACAGTATC
TCAAAAAAGCGATGCCGCGGTTGATCGTTGATTCGTTCGATGATTAAATTCACGGCCAAATGACCGGTCTTTTAGATTACGATC
GTAAACATTACAGAAATGCGTTCACGGAAAATTACAAAATAAATTCTTTCATCCAAATTCAGTTGAATTTTAT[C/T]CAACTCTTTT
ATTCGATGATTTTTTTTAGAGCTTG[G/C]CAGGTTGATCGGAGATGGGTGGAAAGAAGAGGTGTTGCCGTAAGAGCGTTGCGG
CGTGGGAGAAATTGAAATTATTCGGAGGGTGCTCGGATGCGTGGGGCCGGTCCTGAGCCGAGTTGACGTCAGCGGCGCGTG
CGCGCCACAGCGGACGGCACTGCAGAAGCAGGCGAGTTCGCGAGTATATCGATTTTACGATCGTGTCTCTCGCCGCTAGTGT
GTCCGTTTCCTTTAGTTTTTAGTGGCCGTGCTCGTGGTACATGGAGAAAATTGTGAATGGGATTAGTCGCTAGTTGTTTCGCTT
CGTACATCCGCTGACTCGCAACCGTTCGTCACCCTCGCCA

Supplementary Figure 3. Syx1a Upstream SNPs. Each SNP upstream of the start site of *syx1a* is highlighted in bold and yellow. The highlighted SNPs are: LALB_28:262159, LALB_28:263596, LALB_28:265348, and LALB_28:265384, respectively.

>LALB_28:265757-283118

GTGAGTGTCCAAGTAGCCTACGTACAATTCAGCGGATAGACTAAGCCGATTCGCCTAACCG
GAGCATTCGGTCCGCTTGCCAGTCAGTCAGCCAGCCAGCTAGCCTGGCCCGTTAACGTTC
TCTCCCTCCTTCTCTCTCGCCCTCTCTCTTTCCGCCACGATCCTCTTCTCTTTCTTTCCTCG
GCTCGCCATCCGCGTTACTCCTCGTCTCTCGTTAACTCTCGCGGGGACCGCGCGCTTTCT
CTGTCAACTGTCGAAAAATCGGTGCCCCGCGATCACGTCGCCGATCCCCCACGTATATTCT
TTTGTTATGATTCCCCGTGGTATTCTCTGGTCGGTTGCCGTACGTACTTACGTCGAGCGGA
AACGATTCTTCTGCTCGCCGTGACAACTCGTATGCAACTCGTGTGTCGCCTGTTTCTCTTTC
TGTTTCTTTTTGTGGCACACGAGAACGAACGCACACCGTGGCCGTGTGTCTGTGTGCCCG
TTGTCCCTTCGTAAACCGCGCTTCCGCGAGAGATTGATGAGATCGATGCCGGAA**[C/T]**TGA
GGAAAACGAGACCGACAAACAAGAAAGAAGGCGTTACCGAGGTGTCAGAGCCGTTGCGCT
GACTGTCATTCGCGAGCGCGTCCCAGCAGTCGATAGTAGATGGGAGAAGAAAGTCCGTGA
ATCGACGGGAAAAGTGGAAAGATAATTGAACGCGGTGACAGATAGATAGCGGAGACAGAT
ACGTATACAAGTACGTGCATACATATGTACGTATACATATGTATCTTCGTATGCATATATGTA
CAGAGATGGGACAGATATTTGGATAGTTGGATAGATAGAGAAAGGAAAATACGTATGTCCA
TGGATATAGATGGAGTGTCAGCGACGGGGGAGGGGGAGGGACGGGGGAGGGGATAGAG
GGAGAAACCAGTGGCTCCTGTGGCTGACAGGTGTTTGCCAATATATGTTTTTCGTCATCGT
TTATTTCGTAACGATGACAGAATCGCTTGTGACAGCGGCGCGGCTCCCATTTGAACGCGG
ATTTCTTCCCGGATGAGTATGCCAGTCGATTGTTTGGAAGGAACTCCGAGAACGAGAGTAC
CGGCAGCAGTCGATGACTCATACCCGCGTTGACTGACGGCCAAGACAACCGTGGTATTAT
TTGGGCGTATCTGTCGGCGCGGTGCGCATCCGCCATAAATTATTCCTGCATTTTTATCGAC
TTCGCGCGTTCTATGGATCAATTAGACAACGCCGAGTTTCGTCGCTTGTCCTACCGTTCCC
GTTCCGTTCCGGTCCGTTTTACGCTCGGACTTGTCCCGTCGAACCGTCTATCACACGAATC
CACGAAACCACCTGCGGTACCAGCTACGCTGCACGAATTAGTCACACTGACTCGCATAATT
GATCAAACCCCTCGTCCAAACGGAACAACCTTTCCGAAATGGGACGAGTTCAACCGTTCAT
TTCGACTTCTCGAGAAATTCTCGGGTCCAACTTTTGCTCGGAAGTTACGTCTGCGGTTTAA
CACGTTCGCAATGAAGTATTCTTGTAACACCGGCGGTAGGTGCGTGCCGGCGCGAACGTG
TAATGCTCATTTAATATCCATGCAATTCCATGTCACACATATTTTTATTGTATTTCGTTATTTA
TTGAAATATCTGGGTTCCCGACGATTAATAATGTTTATCAGGAGCGGGAAATAAAAGATCAA
CCCTTTGCAACCGAGTGGCGACTCTGAGGCCAAAATTGTTATACCATTATTCAAACTATTTT
GTACATTATCAGTACTTGATAAATTACTAAAAATTTCAGTATTGTACGTGTAAATCAGTTTCG
TATGCATAGAATTAAGAATAATCCTATAAAATGGAAATATTCTAGGTCGGAAGGAAAATTTG
GTATGTACTAGAATGAAAATAA**[A/C]**TCCGAGTGCAAAGGGTTGAAATTGAAAAATTTCACTT
TCTCGATGTACTTGCAAATTAAATGTCATGGAGCTGCGTGTAGAAATTCGCTGCGCGGTTC
CATTTCGCCACAATAATGATTGTTATCGCAATAGTTGAAGATAAAGCAAAGCATATCCTGAC
AATGGCTGGAGGATACTTCGTCCAATATAATTATTTCGTGTATTGTATTCCGGCCTCGACCG
CGACCGCGATCTAGACCGCGATCTAGATCGTGCTCGCGTTCGGCCGGCGTTGCTCGTTCC
TCCGCGTCCTCGATCGTTCTGAATTTCGAGAACTCGATCTCGTTGAGCCTCTCTTTCGACC
GTGTAAATCACGGGCCGACTTCCATCCATTCCGACAATTGCGATCCGCGTTCCAATTCTCT
CGAAATCGTAGCGTGGGAATACGTCGCGTACAGTAATTCCGGTCGGGACGACACGGTCTC
GCATATTACGTCAAAAATATATTCCAATTTCCGGTAACAGGTTGGCTGATAAAATCCTGCAG
ACCATCCCTTGAAATTAATTTTTTACCGGCACACGTAACGTCTTCCCGTACAAGTCGGTCTC
GCAACGTGTTAATTATTCTTTTGATGTATGTTACCCGAGAAATTAAGTAATGTTTGTTAAATA
GAATTACTCCCTCGCGTGGAGGAAGCAGATGTATCCGTTCTGCTTGATTATTAATATGTCG
AAGACTCTGCGGAACTTTAGAGGTTAATCCAACCCCCAGGATGCCATTTAGAAAAGGTTAC
AATTTTGATAAAATCTTAACAAAGAGCCGT**[A/G]**GAAGAGCAAGATCTCGAGTCGGGAGAAT
CAAAAGGACTAAAACGTGACAAAAAATTGAGGCGGACCGAAATGTAATTGGGCCACGACAT
CCTGAATCGTGAGTCGTCTCATCGGGATGATCCGGATTAGATGGCCTTATGCATGGGATG

Supplementary Figure 4. Syx1a Intronic SNPs. Each SNP located in the intron *syx1a* is highlighted in bold and yellow.

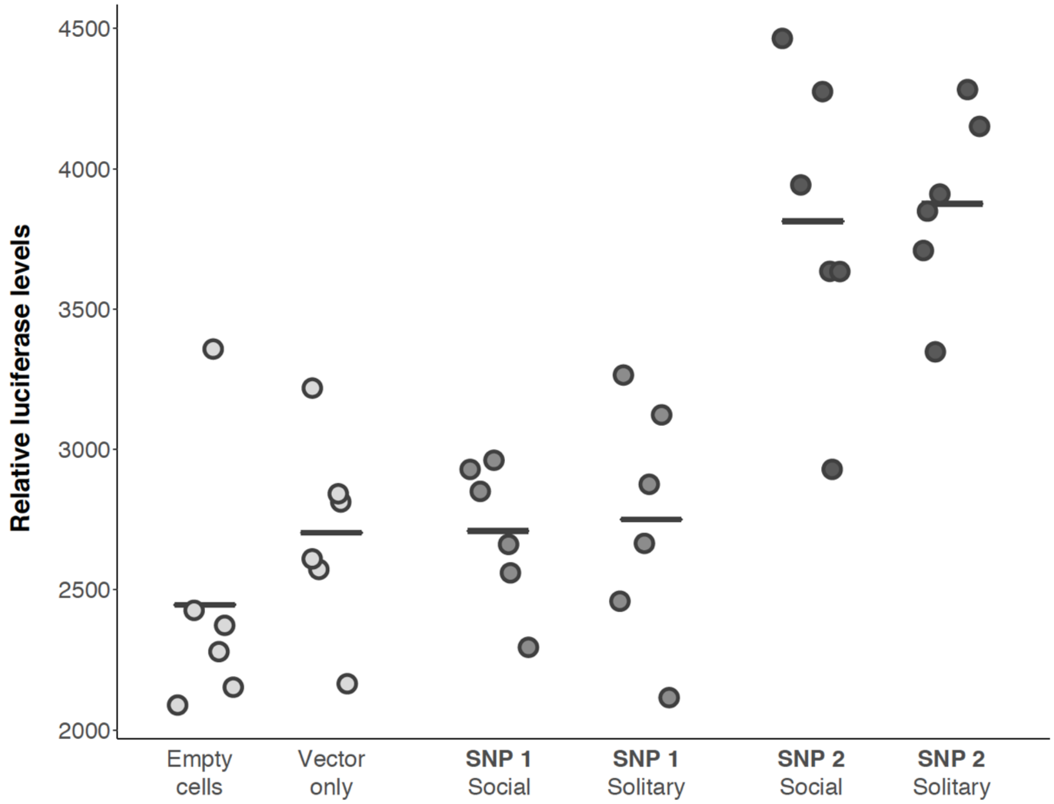

Supplementary Figure 5. Luciferase reporter assay for promoter activity at SNPs 1 and 2. Both SNP 2 alleles produced higher levels of reporter gene expression than the controls: empty cells or vector only constructs (Tukey's honestly significant difference (HSD) post-hoc test, $p<0.001$ in all comparisons). Reporter expression levels did not differ between social and solitary alleles at either SNP (Tukey's honestly significant difference (HSD) post-hoc test; SNP, 1 p=1.0; SNP 2, p=0.98).

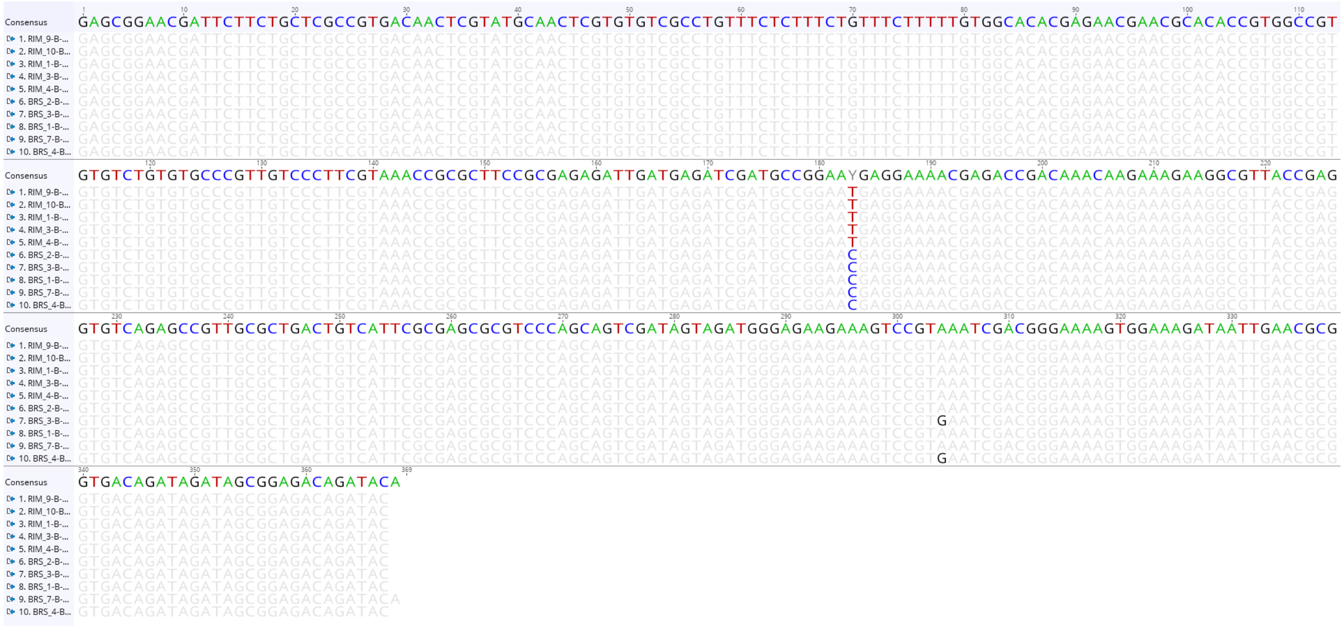

Supplementary Figure 6. Genotypes at SNP 2 for the social and solitary individuals included in the qRT-PCR analysis. Individuals from the social population carried a “T” at LALB_28:266299, while individuals from the solitary population had a “C”. All individuals were haploid males.
