# Supplementary material for: The genetic basis of a social polymorphism in halictid bees
Source: Nat Commun. 2018 Oct 18;9:4338. doi: 10.1038/s41467-018-06824-8 (PMC6194137; doi:10.1038/s41467-018-06824-8)
Supplement: Supplementary file 2 — Description of Additional Supplementary Files [file 41467_2018_6824_MOESM2_ESM.pdf]

Description of Additional Supplementary Files

File Name: Supplementary Data 1
Description: Samples used in this study. Each row represents an individual sequencing library. Columns are as follows: name of the sample as uploaded to SRA; plate name of the sample; well number of the sample (currently archived in the Kocher Lab); SiteName=name and date of site where sample was collected; population name of each sample; region in France where population is located; behavioral type of that population; sex of the individual bee that was sequenced; latitude, longitude of collection site; number of reads generated from the sequencing library; and the average sequencing depth for the sample.

File Name: Supplementary Data 2
Description: A total of 194 SNPs passed a genome-wide significance threshold of $5x10^{-5}$. Columns A-L represent output from GEMMA analysis; see manual for further details. FST was calculated between social and solitary populations. “Social a1 freq” and “Solitary a1 freq” contain the allele frequencies for the alternate alleles in social and solitary populations, respectively. Columns P, Q, R, and S are functional annotations output by SNPeff. Lalb_v3 is the gene name in the genome assembly included in this manuscript. SwissProt assignments were identified using trinotate. Lalb_v2 provides the corresponding gene name from the previous genome assembly if available.

File Name: Supplementary Data 3
Description: Gene ontology categories over- and under-represented by candidate loci. Please see (Supek et al 2011) for detailed description of output.
